# Implementation analysis of a case management intervention for people with complex care needs in primary care: a multiple case study across Canada

**DOI:** 10.1186/s12913-023-09379-7

**Published:** 2023-04-19

**Authors:** Catherine Hudon, Mathieu Bisson, Maud-Christine Chouinard, Alannah Delahunty-Pike, Mireille Lambert, Dana Howse, Charlotte Schwarz, Olivier Dumont-Samson, Kris Aubrey-Bassler, Fred Burge, Shelley Doucet, Vivian R. Ramsden, Alison Luke, Marilyn Macdonald, André Gaudreau, Judy Porter, Donna Rubenstein, Cathy Scott, Mike Warren, Linda Wilhelm

**Affiliations:** 1 Department of Family Medicine and Emergency Medicine, University of Sherbrooke, Pavillon Z7-Room 3007, 3001, 12E Avenue Nord, Sherbrooke, QC J1H 5N4 Canada; 2 Centre Hospitalier Universitaire de Sherbrooke Research Centre, Sherbrooke, QC Canada; 3 Faculty of Nursing, University of Montreal, Montréal, QC Canada; 4 School of Nursing, Faculty of Health, Dalhousie University, Halifax, NS Canada; 5 Primary Healthcare Research Unit, Memorial University, St-John’s, NL Canada; 6 Department of Nursing and Health Sciences, University of New Brunswick, Fredericton, NB Canada; 7 Department of Academic Family Medicine, University of Saskatchewan, Saskatoon, SK Canada; 8 Diabetes Action Canada, Toronto, ON Canada; 9 Nova Scotia Health Authority, Halifax, NS Canada; 10 Patient Advisors Network, Toronto, ON Canada; 11 Canadian Cancer Society, Toronto, ON Canada; 12 Patient Advisory Council, Newfoundland and Labrador SPOR SUPPORT Unit, St. John’s, NL Canada; 13 Canadian Arthritis Patient Alliance, Ottawa, ON Canada

**Keywords:** Case management, Frequent users, Implementation, Multiple case study, Primary care, Chronic conditions, Complex care needs

## Abstract

**Background:**

Case management is one of the most frequently performed interventions to mitigate the negative effects of high healthcare use on patients, primary care providers and the healthcare system. Reviews have addressed factors influencing case management interventions (CMI) implementation and reported common themes related to the case manager role and activities, collaboration with other primary care providers, CMI training and relationships with the patients. However, the heterogeneity of the settings in which CMI have been implemented may impair the transferability of the findings. Moreover, the underlying factors influencing the first steps of CMI implementation need to be further assessed. This study aimed to evaluate facilitators and barriers of the first implementation steps of a CMI by primary care nurses for people with complex care needs who frequently use healthcare services.

**Methods:**

A qualitative multiple case study was conducted including six primary care clinics across four provinces in Canada. In-depth interviews and focus groups with nurse case managers, health services managers, and other primary care providers were conducted. Field notes also formed part of the data. A mixed thematic analysis, deductive and inductive, was carried out.

**Results:**

Leadership of the primary care providers and managers facilitated the first steps of the of CMI implementation, as did the experience and skills of the nurse case managers and capacity development within the teams. The time required to establish CMI was a barrier at the beginning of the CMI implementation. Most nurse case managers expressed apprehension about developing an “individualized services plan” with multiple health professionals and the patient. Clinic team meetings and a nurse case managers community of practice created opportunities to address primary care providers’ concerns. Participants generally perceived the CMI as a comprehensive, adaptable, and organized approach to care, providing more resources and support for patients and better coordination in primary care.

**Conclusion:**

Results of this study will be useful for decision makers, care providers, patients and researchers who are considering the implementation of CMI in primary care. Providing knowledge about first steps of CMI implementation will also help inform policies and best practices.

**Supplementary Information:**

The online version contains supplementary material available at 10.1186/s12913-023-09379-7.

## Background

Eighteen percent of people followed in primary care, where many of their health care needs are provided [1–3], have complex needs [4] such as multiple chronic conditions, mental health, and socioeconomic challenges [5]. They often face multiple barriers to accessing appropriate and coordinated care, increasing their risk of becoming frequent users of healthcare services [6, 7]. With increased complexity of people’s needs, comes decreased care accessibility and an increased risk of fragmented care, putting these people at increased risk of poorer outcomes: decreased quality of life, and increased disability and mortality risk [8]. Complex needs call for integrated care across providers and sectors [9].

Different healthcare interventions have been proposed to improve integrated care and mitigate the negative effects of complex needs on patients, primary care providers and the health system, including case management interventions (CMI), individualized services plan, patient education and counseling, problem-solving, and information sharing. Defined as a collaborative approach that assesses, plans, facilitates, and coordinates care to meet patient and family health care needs [10, 11], case management is the most frequently performed intervention [12–14]. Research has demonstrated that CMI improve patient's integrated care experience and promote a better utilization of health care resources, reducing emergency department (ED) visits, hospitalization rates, and health care costs [12–15].

Reviews have addressed factors influencing CMI implementation and reported common themes related to the case manager role and activities, collaboration with other primary care providers, CMI training and relationships with the patients [16–21]. However, some of these reviews highlighted the heterogeneity of CMI assessed across studies, targeting various populations such as frail elders, psychiatric, substance abuse patients or clients with cognitive impairments. The reviews also reported on the variety of settings within which CMI had been implemented. Better understanding the implementation of CMI in primary care may increase its transferability. In addition, the first steps of implementation such as preparing change at the organizational and individual levels, engaging stakeholders, and following an implementation plan have been identified as critical activities for a successful implementation [22]. These steps need to be further assessed for implementation of CMI in the context of primary care [22–24].

This study aims to identify facilitators and barriers to the first implementation steps of a CMI in primary care for people with complex care needs who frequently use healthcare services.

## Methods

### Design and context of the study

A qualitative multiple case study using a participatory approach [25] was conducted to provide an in-depth description of the facilitators and barriers of the CMI implementation. This design is useful to furthering develop understanding of complex interventions and how they are influenced by different contexts, and environments [26]. The current implementation analysis was the first phase of the PriCARE research program, the second being a realist evaluation within the same clinics [27]. The goal of PriCARE [27] is to study the implementation of a CMI for people with complex care needs who frequently use healthcare services in primary care clinics in five Canadian provinces: New Brunswick, Newfoundland-and-Labrador, Nova Scotia, Quebec, and Saskatchewan. In accordance with the Canadian Strategy for Patient-Oriented Research [28], the study was framed within the patients’ priorities. As full members of the research team, patient partners participated in every step of the study and contributed to the nurse case managers training. Decision makers and clinicians also collaborated with the research team at different levels according to their availability and expertise, mainly for the implementation of the CMI [25]. The participatory approach of the PriCARE research program, including the governance structure, are detailed elsewhere [25].

### The case management intervention (CMI)

In line with North American guidelines [10, 11] and findings of prior studies [12, 14, 29–31], including some authored by members of this study team [32, 33], the CMI consisted of four main components: 1) assessment of patient needs and preferences; 2) co-development and maintenance of a patient-centred individualized service plan, i.e. a plan co-created with the patient, family and other partners to coordinate all the services required to meet the patient’s life plan (goals and desired outcomes); 3) coordination of services among all partners; and 4) education and self-management support for patients and families. The CMI was delivered by primary care nurses, either based at the clinic or hired for the position, over a 12-month period. Nurse case managers were provided with three to six hours of training to lead and coordinate case management activities 6–8 h a week within the primary care clinic. They also participated in a community of practice to discuss questions and challenges. Each clinic was to recruit 30 individuals meeting the following criteria (i.e. the final criteria after consultation with primary care providers): frequently accessing health care (primary health care service, ≥ 4 ED visits and/or hospitalizations in the previous year) [5, 34], living with at least one chronic condition identified by Bayliss et al., [35] having a score ≥ 19 on the INTERMED-Self-Assessment Questionnaire (evaluating complex healthcare needs) [36] and deemed to benefit from the CMI in the opinion of the primary care providers (i.e. nurse case managers and family physicians). Patients excluded from the study were frail elderly with loss of autonomy, and patients with a life-expectancy of less than one year. Nurse case managers offered the CMI to patients over a 12-month period. The CMI assessed in this study is detailed elsewhere [37].

### Conceptual framework

Data collection and analysis relied on a conceptual framework for the implementation of a CMI by Danish et al. [37]. This framework was inspired by two multilevel conceptual frameworks. The first is the Rainbow Model for Integrated Care of Valentijn et al. [38], which combines the concepts of primary care and integrated care. This model includes six dimensions: clinical, professional, organisational, systemic, functional, and normative integration. The second is the Consolidated Framework For Implementation Research of Damschroder et al., [22] which aims to foster the implementation of complex and multilevel interventions in healthcare. This framework provides a taxonomy of constructs categorized in five domains that can be used across a variety of contexts: intervention characteristics; outer setting; inner setting; characteristics of the individuals involved; and process of implementation.

To properly address the first steps of CMI implementation, emphasis was placed on the first three constructs of the implementation process: [22] planning, engaging, and executing. Planning refers to “the degree to which a scheme or method of behavior and tasks for implementing an intervention are developed in advance and the quality of those schemes or methods”. Engaging refers to the involvement of the right people in the implementation and the use of a combined strategy of social marketing, education, role modeling, training, and other similar activities. Executing refers to the implementation being caried out according to the plan developed. When implementing these dynamic, non-sequential and non-linear activities, facilitators and barriers may arise at multiple levels of the intervention delivery [22, 37].

### Data collection

#### Sampling and description of the clinics

Six primary care clinics in four provinces in Canada were recruited using a purposeful sampling strategy [39], with each CMI implemented in the clinic considered a case. The researchers' knowledge and experience within the health network helped them identify the clinics. Inclusion criteria for the clinics were: no CMI had been previously implemented, strong interest and engagement of health services manager and clinical team, and availability, and strong interest of a primary care nurse to act as case manager. The clinics were described based on the following characteristics: location (Canadian province), population (number of inhabitants) of the city where the clinic is located [40], urban, semi-urban or rural area [41], date of creation of the clinic (year), university affiliation (yes/no), composition of the staff (nurse case managers, physicians, other nursing staff, other health care providers), number of registered patients, and mode of physician remuneration. Most of the information was collected directly from the clinics.

#### Sampling of the participants

Key informants were recruited through purposeful sampling [39] in each of the six participating clinics, and contacted by telephone or email by the local research coordinators in charge of data collection.

#### Semi-structured interviews and focus groups

Semi-structured interviews and focus groups were conducted by one interviewer of the research team (MB, MC, BC, ODS, DH, ML) between January 2019 and January 2020 with the staff of each clinic, including health services managers, nurse case managers, family physicians, and other health professionals. The interview guide was composed of 14 open-ended questions based on the conceptual framework to elicit information related to each of the implementation analysis elements, including planning, engaging and executing phases of the implementation, as well as the contextual elements that influenced them [37]. The interview guide is available in Additional file 1. All interviews and focus groups were audio-recorded, conducted either over the telephone or in person at the clinic, took between 45–60 min to conduct and were transcribed verbatim.

#### Field notes

Field notes were written by research team members (MB, MC, ADP, ML, DH, CS) during the same 12-month period that the interviews and focus groups were conducted. They included reflections about meetings with different stakeholders such as: nurse case managers, health services managers and patient partners; research activities; and the challenges of implementing CMI in different primary care settings across Canada.

### Thematic analysis

A mixed thematic analysis was used based on the conceptual framework (deductive) and emerging themes (inductive) as proposed by Miles, Huberman and Saldana [42]. In line with the objectives of the research, a codebook was developed by the research team (MB, MC, BC, AD, ODS, DH, ML) based on the categories of the conceptual framework, including planning, engaging and executing steps of the implementation, as well as contextual elements that influenced them [37]. The research team met several times to validate the codebook by coding the first transcripts. Once the coding was consistent, the rest of the transcripts were coded. All qualitative data including field notes were integrated and analysed in NVivo V.12 server software (QSR International Pty). For each case, themes from the conceptual framework and emerging themes (including facilitators and barriers to CMI) were listed and discussed during regular team meetings with the researchers, research coordinators and assistants, and patient partners to identify the more salient characteristics of the CMI within each clinic. Themes were incorporated into a descriptive and interpretative matrix [42], which was revised following an iterative process and validated by all team members. Themes and their meaning were drafted using an iterative process until consensus was reached. Two cross-case tables (Tables 4 and 5) were created aiming to distinguish facilitators and barriers to the CMI implementation: one related to the contexts of the clinics, the other related to intervention characteristics. The data were used to contextualize the implementation of the CMI in each clinic in the form of case stories. A table (Table 3) was created for this purpose to facilitate understanding of the implementation process. Patterns and contrasts were consciously searched and identified [26]. According to the participatory approach [25], all research team members, including patient partners, participated in key steps of the analysis [43].

Several strategies were applied in this qualitative study to ensure trustworthiness, which includes credibility, dependability, confirmability and transferability [44]. The participatory approach ensured meaningful interpretation, credibility and reliability [44, 45]. Field notes were used to track the data collection process and analysis to ensure dependability [44]. The research team promoted several triangulation techniques (methodological, data source and investigators), ensuring confirmability [43, 44]. Discussions and interactions within the team to examine the research process and interpretation of findings also ensured reflexivity and confirmability [44]. In-depth description of the context of each case using various qualitative methods ensured transferability [44].

This study received approval from Ethics Review Boards in each of the 4 participating provinces: Comité d’éthique du Centre intégré universitaire de santé et services sociaux de l’Estrie – Centre Hospitalier Universitaire de Sherbrooke; Research Ethics Boards Horizon Health Network; University of New Brunswick Research Ethics Board, Research Ethics Board Memorial University; Nova Scotia Health Research Ethics Board. Informed consent was obtained verbally, or in written form, and was documented by a member of the research team.

## Results

### Description of clinics and participants

Table 1 contains descriptive characteristics of the clinics where the CMI was implemented.

**Table 1:** Characteristics of the clinics where the CMI was implemented

Characteristics	Clinics					
	A	B	C	D	E	F
Location (Canadian province)	Quebec	Quebec	Nova Scotia	Nova Scotia	New Brunswick	Newfoundland and Labrador
Population (number of inhabitants) of the city where the clinic is located^a^	167 162	60 077	6 700	1 330	67 575	116 895
Urban, semi-urban or rural area	Urban	Semi-urban	Rural	Rural	Urban	Urban
Date of creation of the clinic	1985	2002	2020^b^	2018	2002	1972
University affiliation	Yes	No	No	No	No	Yes
Staff						
NCMs	4	2	1	1	2	1^c^
Family physicians	30	16	2	4^c^	8	14
Other nursing staff^d^	15	3	1	2	15	2
Others	13	6	3	5	12	6
No. of registered patients	16 487	23 299	2 200	3 036	12 000	6 620
Mode of physician remuneration	Hourly	Fee-for-services	Fee-for-services	Hourly	Hourly	Hourly

*NCM* nurse case manager

^a^The population served by each clinic may extend beyond the city’s territory

^b^Created in 2003 but moved to a new town in 2020

^c^At the time of the data collection

^d^Includes nurse practitioners, registered nurses and licensed practical nurses

Table 2 contains the characteristics of the participants. Individual interviews were conducted with nurse case managers (*n* = 10), health services managers (*n* = 5), a family physician and other health care providers (*n* = 4). Six focus groups were conducted with family physicians (*n* = 20), and other health professionals (*n* = 8). Participants self- identified as either male (*n* = 6) or female (*n* = 42), between the ages of 25 and 74. Their years of professional experience ranged from 1 to 42 (mean = 13) and years of experience in their clinics from 1 to 32 (mean = 8) years.

**Table 2:** Characteristics of the participants (*n* = 48)

**Variables**	
Gender: n (%)	
Female	42 (88)
Male	6 (12)
Age (years): n (%)	
25–44	30 (63%)
45–64	17 (35%)
65 +	1 (2%)
Profession: n (%)	
Health service managers	5 (10%)
NCM	10 (20%)
Family physicians	21 (44%
Other primary care providers	12 (27%)
Years practicing: mean	13
Years within the organization: mean	8

*NCM* nurse case manager

### Case stories

Table 3 presents the case stories, i.e. how the first steps of the CMI implementation took place in each clinic. This includes similarities and differences in contextual factors such as organizational characteristics of the clinic, experience, skills, and background of the nurse case managers, main collaborations and activities related to the implementation of CMI, and other information specific to the early implementation process (e.g., particularly relevant facilitators and barriers.

**Table 3:** First steps of CMI implementation in each clinic – Case stories

Case	Description
**A**	The services of this family medicine group ^a^ are provided in two sites located in a medium-sized city. The first site is close to a lower-income neighbourhood and adjacent to a local community health centre ^b^. The second site is smaller and further away from the downtown area than the first. The four NCMs in both sites have good team cohesion, trusting relationships, strong leadership, and significant professional experience (e.g., case management, chronic illnesses). They divide their responsibilities equally, including case management activities, thus strengthening teamwork and collaboration. They also have strong collaboration with the clinic’s family physicians who introduce them to patients who may benefit from the intervention. In this clinic, a community of practice allows NCM to reflect on their roles, solutions to common issues, and concerns regarding inclusion criteria. The community of practice provided support from two experienced nurses in case management and from a researcher patient partner on how to approach patients
**B**	This clinic includes seven sites, two of which are involved in the CMI. One site has experienced more nursing staff turnover than the other. The two NCM in both sites who lead the intervention have limited experience in primary care. Prior to starting the CMI, both had experience recruiting patients in a research project, and one had previous experience with ISP. This NCM also has a good working knowledge of health and social resources, which helped facilitate organizational collaboration. The NCM work collaboratively with the nurse practitioner and family physicians at the clinic. The staff showed leadership during the first steps of the CMI implementation, and the health services manager supported them. The clinic also has support from an experienced hospital NCM who has access to patient health information. There were several meetings with the hospital NCM, the clinic’s family physicians and the clinic’s manager to provide an overview of the project, plan the next steps and clarify their roles
**C**	This clinic is located in a town in a rural area. The NCM is an experienced family practice nurse who worked as a critical care nurse at the local hospital in the adult medical-surgical intensive care unit. This individual was chosen for this role by the health services manager for the region. The NCM works collaboratively with the nurse practitioner and family physicians at the clinic. Collaboration between the NCM and health services manager is strong, with frequent discussions about identifying and recruiting patients as well as strategies for patient meetings, including the ISP. During the implementation stage, the NCM and health services manager performed a mock recruitment and consent to develop confidence and build research skills. There were organizational meetings with clinic family physicians, the nurse practitioner and staff to plan for the CMI implementation. There was also a meeting to discuss patients who could benefit from the CMI. The NCM took ownership of their role and would be a very good mentor for others coming into a NCM role in the region or across the province
**D**	This clinic is adjacent to a local hospital. The NCM is an experienced family practice nurse working in the clinic since it opened (early 2018), with previous experience working in the local hospital in the ED and on the medical floor. This individual was chosen for this role by the health services manager for the region, who also acts as the clinic manager. The NCM works collaboratively with other providers, such as the nurse practitioner and family physicians at the clinic, which helps to foster and support understanding of the CMI within the clinic. Collaboration between the NCM, clinic manager and regional primary healthcare coordinator is frequent and open. In particular, the NCM and primary healthcare coordinator worked together at the implementation stage to identify participants and set up systems to keep track of potential and recruited patients. There were several meetings with clinic family physicians, the nurse practitioner and staff to discuss patients who would be a good fit for the CMI, based on intervention inclusion and exclusion criteria. A meeting was held at the clinic to discuss barriers to patient participation, which centred around a discussion on solutions for these barriers
**E**	The clinic is adjacent to an outpatient facility which houses medical clinics, diagnostic services, and an urgent care centre. The centre provides several services and primary health care programs, while offering additional satellite centres in priority neighbourhoods. Leading the CMI implementation, the NCM hails from the adjoining community health centre and is knowledgeable about the health system. This individual has been chosen due to a strong medical background and knowledge of the healthcare system. The NCM is highly experienced in community partnership initiatives aimed at addressing social determinants of health (e.g., food security and advocacy for affordable housing). The NCM works collaboratively with the health service manager, where a positive history of working together seems to be helping to move the project along. In the clinic the NCM was physically separated from the primary care providers. The CMI is strongly supported by management and the team of primary care providers. There is regular access and communication within the clinical team
**F**	This clinic is near a hospital and medical school. Many of its patients receive health and social supports through an associated clinic, but the clinic largely relies on referrals to external supports to meet patients’ complex needs. A NCM external to the clinic was hired to steer the CMI. Because the NCM was outside the patient’s existing circle of care, the family physician introduced the NCM in order to foster a relationship of trust. The NCM brings to the CMI valuable experience and skills, as well as an established network of local health providers and social supports. This helps to connect with and support patients and to engage and coordinate health providers and social supports across multiple organizations and physical locations. The NCM works collaboratively with family physicians, who may share important patient information using the clinic’s electronic medical record (EMR) system

*CMI* Case management intervention, *ED* Emergency department, *EMR* electronic medical record, *ISP* Individualized services plan, *NCM* Nurse case manager(s)

^a^In the province of Quebec, a family medicine group is a primary care clinic including family physicians who work together and in close collaboration with other health and social services providers (e.g., nurse, social worker, pharmacist, psychologist, etc.)

^b^In the province of Quebec, a local community health centre is a public organization offering front-line health services and assistance programs to the population

### Facilitators and barriers to the first steps of the CMI implementation in primary care clinics

The thematic analysis allowed 13 themes describing the facilitators and barriers to the first steps of the CMI implementation to be identified across the six primary care clinics. The first four themes are clinic/context specific, and the nine other themes are intervention specific. Tables 4 and 5 portray the themes by clinic and intervention respectively.

**Table 4:** Facilitators ( +) and barriers (-) to the first steps of the CMI implementation – Contexts of the clinics

**Themes**	Case					
	**A**	**B**	**C**	**D**	**E**	**F**
**Roles and interprofessional collaboration**	( +) Formal collaborative approach ( +) NCM autonomy and leadership ( +) Informal connections among providers	( +) Formal collaborative approach ( +) NCM autonomy and leadership	( +) Formal collaborative approach ( +) Informal connections among providers	( +) Formal collaborative approach	( +) Excellent communication within the clinic staff ( +) Informal connections among providers (-) Difficulty contacting specialist physicians	( +) Formal collaborative approach and team meetings where clinic providers discuss complex patients (-) Few psychosocial resources within the clinic
**Organizational collaboration**	( ±) Positive relationships with many different organizations, but lack of collaboration with some (-) Increasing reduction in services offered from other organizations	( ±) Positive relationships with many different organizations, but lack of collaboration with some (-) Long delay before the take-over of patient care from other organizations	( ±) Positive relationships with many different organizations, but lack of collaboration with some particularly mental health (-) Challenges accessing some community resources available to clinic	( ±) Positive relationships with many different organizations, but lack of collaboration with some, particularly mental health (-) Lack of access to resources	( ±) Positive relationships with many different organizations, but lack of collaboration with some, particularly mental health ( +) Access to various services in close proximity (-) Inability to share records with external departments	( +) External organizations help to overcome the lack of psychosocial resources (-) Lack of collaboration with some organizations (-) No simple stream-lined process for connecting to community supports
**Access to patient health information**	( ±) Affiliation to regional health centre gives easier access to EMR, but access is still limited (-) Regulations surrounding confidentiality may interfere with the exchange of information among providers	(-) Lack of access to EMR and limited information sharing (-) Regulations surrounding confidentiality may interfere with the exchange of information among providers	(-) Lack of access to EMR	( ±) Systems and tools exist for specific medical information sharing, but more tools are needed (-) Systems are not used by all providers	( +) Ability to share records between departments ( ±) Systems and tools exist for specific medical information sharing, but more tools are needed (-) Regulations surrounding confidentiality may interfere with the exchange of information between providers	( +) Systems and tools exist for specific medical information sharing (-) Communication from referrals not consistent
**Patient-centered care**	( +) Patient centeredness around building a strategy together (-) Goals of care not always discussed with patients	(-) Need for more training in patient self-management support for NCMs	( +) Patient centeredness around building a strategy together ( +) Having patients self-identify which services or providers they need	( +) Patient centeredness around building a strategy together ( +) Empowering patients to self-manage	( +) Access to translation services (-) Literacy and language constraints	( +) Establishing trusting relationship with patients and staying in touch regularly ( +) Empowering patients to self-manage

**Table 5:** Facilitators ( +) and barriers (-) to the first steps of the CMI implementation – Characteristics of the CMI

**Themes**	A	B	C	D	E	F
**Clinical and managerial support for CMI**	( +) Nursing and medical leadership	( +) Nursing, medical, and managerial leadership ( +) Group discussion with clinical team and clinic manager about scheduling and individual involvement	( +) Group discussion with clinical team and clinic manager about scheduling and individual involvement	( +) Managerial leadership ( +) Clinic manager introduced CMI to clinical team	( +) Managerial leadership	(-) Difficulty in coordinating multiple supports that are physically located in several places
**Capacity development (including team)**	( +) Pre-existing community of practice	(-) Lack of information to clinical team members about CMI	( +) Team meetings to discuss CMI planning and organization	( +) Team meetings to discuss CMI planning and organization ( +) Pre-existing team meetings to discuss complex patients (-) More training and support needed for NCM role	(-) More training and support needed for CMI and NCM role (-) NCM does not work within the clinic	( +) Pre-existing team meetings to discuss complex patients (-) NCM does not work within the clinic
**Experience of NCMs**	( ±) Experienced and skilled NCM, but needed support to start the CMI ( +) CMI activities already performed	( +) Case management activities already performed (-) NCM lack of knowledge of certain chronic diseases	( +) Experienced NCM	(-) NCM needed support to feel comfortable in CMI	( +) Experienced and skilled NCM	( +) Experienced and skilled NCM
**Time required**	(-) Time consuming, work overload	(-) Time consuming, work overload (-) Concern about being overburdened with complex cases	(-) Time and resources consuming ( ±) Information gathering is considerable upfront, but useful investment of time	(-) Time consuming, work overload ( ±) Information gathering is considerable upfront, but useful investment of time	(-) Time consuming, work overload	(-) Referring the patient to other services may introduce delays and missed appointments and is time consuming
**Identifying patients**	( +) NCM planned to identify patients with the help of family physicians (-) Recruitment criteria not aligned with what providers had anticipated	( +) Involvement of other providers to create list of frequent users (-) Difficulty finding the appropriate patients (-) Recruitment criteria not aligned with what providers had anticipated	(-) Difficulty finding the appropriate patients (-) Recruitment criteria not aligned with what providers had anticipated	(-) Difficulty recruiting patients (time consuming)	( +) NCM and family physicians identified patients (-) More guidance required for patient recruitment	( +) Family physicians identified targeted patients (-) Steps to identify high needs patients necessary but challenging in a clinical setting
**Recruiting/enrolling patients**	( +) Family physicians introduced NCM to patients to foster trust (-) NCM concerns about first contact with patients and recruitment	( +) NCM confidence about first contact with patients	(-) NCM concerns about first contact with patients and recruitment and consent process	(-) NCM concerns about first contact with patients and recruitment and consent process	(-) NCM concerns about first contact with patients and recruitment	( +) Family physicians introduced NCM to patients to foster trust
**ISP**	(-) NCM concerns about ISP planning and organization	(-) NCM concerns about ISP planning and organization	(-) Concern about difficulty in assembling all of the providers together for ISP	( +) Team brainstorming about best way to do ISP, potential debriefing after the first one	(-) Concern about difficulty in getting all of the providers together	(-) Risk of providers not having interest or time to engage
**Patient engagement**	(-) Providers’ concerns about motivating/ mobilizing patients with complex needs (-) Concern about the targeted “complex” clientele	(-) Providers’ concerns about motivating/ mobilizing patients with complex needs (-) Need for more training of NCM in self-management support		(-) Provider concern about time commitment for patients with complex needs ( ±) Concern about the self-management support for patients, but the ISP could help	( +) Access to translation services (-) Providers’ concerns about motivating/ mobilizing patients with complex needs	( +) Fostering patient commitment to intervention
**Provider perception of CMI**	( +) Positive perception from family physicians and NCM ( +) Similar to what clinic was doing, but CMI is more organized ( +) Individual approach with patients is more appropriate than group approach (-) Concern about potential confusion due to duplication with other programs	( +) Positive perception in general from other primary care settings (-) Physician skepticism (-) Concern about potential confusion due to duplication with other programs	( +) NCM sees CMI as natural fit ( +) Similar to what clinic was doing, but CMI is more organized, streamlines what is needed (-) Concern about how patients will experience this new way of doing things	( +) Comprehensive approach to care ( +) More resources for patients (-) Concern about implementing the intervention (-) Provider skepticism	( +) Research team support ( +) Adaptability of CMI ( ±) NCM had positive perception, but felt pressure due to work overload (-) Physician skepticism	(-) Difficult for family physicians to hand over CMI-related tasks (-) Physician skepticism and concern about missing out on their patients’ care ( ±) Concern about patients’ adherence to CMI

#### Themes related to context of the clinics (Table 4)

In most cases, there was good interprofessional and organizational collaboration before the CMI implementation characterized by proximity among the providers as well as the availability of a variety of services. However, collaboration with certain external organizations remained difficult, specifically mental health resources and community-based organizations. Access to patient health information, specifically patient hospital records and charts, was problematic across cases. Lack of access to electronic medical records (EMR), lack of adequate systems and tools, and rules related to confidentiality were barriers to information sharing. However, some clinics shared tools amongst organizations, such as writing tasks or notes within the EMR, which facilitated access to information. Finally, a patient-centred and self-management approach was commonly promoted in the clinics, which facilitated the implementation of the CMI, especially when the patient was included in the communication with providers.

#### Themes related to characteristics of the CMI (Table 5)

Leadership of the primary care nurses and family physicians as well as the health service managers, who were generally open to, and had a positive perception of the CMI, facilitated its first steps of implementation. The experience and skills of the nurse case managers as well as capacity development within the team, were also facilitators to the early implementation. However, the dedicated time required to implement CMI for the nurse case managers was a common issue in all cases and raised concern of increased workload in the short term, even if it was seen as a useful investment of time later. Since the initial recruitment criteria were not aligned with what nurse case managers and family physicians had anticipated, more flexible criteria were selected before patients’ recruitment, particularly the inclusion of patients who would benefit most from CMI, based on professional judgment. All nurse case managers expressed apprehension around organizing meetings between multiple health providers with each patient for the individualized service plan, apart from one clinic. In this case, the health care team brainstormed the best method for conducting the individualized service plan and decided on debriefing with providers after the first was completed to engage them and have their concerns, if any, addressed. Participants raised concerns regarding motivating and mobilizing patients with the most complex needs. Participants considered organizing team meetings within the clinic and building a community of practice for the nurse case managers at the beginning of the implementation process as an opportunity to address primary care providers’ concerns with emphasis on patient identification, recruitment, and tools and resources required for CMI activities. Despite time concerns, CMI was described as a comprehensive, adaptable, and organized approach to care that provides increased resources for patients.

## Discussion

This study identified barriers to and facilitators of a CMI implementation for patients that frequently use health care services across multiple primary care clinics in Canada. It provides new knowledge that focuses on the first steps (planning, engaging, and executing) of implementation of such an intervention, helping researchers, health services managers, and primary care providers to identify – and mitigate – potential barriers at this critical early stage.

In this study, primary care providers were actively involved in targeting patients they deemed most likely to benefit from the intervention. The findings indicate that the engagement of nurse case managers and family physicians increased when they combined administrative/health care data with professional judgement, as suggested in the literature for case finding [29, 46, 47]. Two qualitative studies on healthcare professionals' perspective of case finding for patients with chronic conditions in the UK, reported similar this relationship between engagement and case finding [48, 49]. In line with the literature, results from the current study demonstrate that establishing a strong communication plan, and buy in from the whole team, help enhance stakeholders’ understand their roles and increase engagement in CMI [16, 19, 22, 50, 51]. Strong leadership and active support from primary care providers and health services managers increased stakeholders and nurse case managers engagement, facilitating the implementation of the intervention. These factors and their respective impacts are noted in the literature [52–54]. The selection of skilled nurse case managers also helped facilitate the implementation of a successful CMI initiation. This is supported by other studies where the importance of case managers’ leadership [54], autonomy [16], good communication practices [16, 17], interpersonal relationships [19, 29], self-management support, problem-solving and negotiation capacities, and brokerage skills are discussed [29]. The findings from the present study suggest that in addition to identifying case managers with the skills mentioned above, participating in a community of practice, which allowed nurse case managers to share information, experiences and learnings, may be useful. In a primary care practice model for caring for patients with multimorbidity, Soubhi et al. highlight that communities of practice can improve care for patients with multiple conditions through an adaptive and iterative process of collective knowledge management, gain of insights, and development of new care strategies [55].

It was difficult for nurse case managers to access and share patient health information, which acted as a barrier to the setting up the intervention. Standardized methods of data entry, such as the use of electronic medical records for developing, maintaining, and accessing patient care plans could facilitate the implementation of CMI [16, 52]. However, even if shared health information technology improves care access, quality, and coordination, and decreases the costs of care [56–60], it may conflict with the ethical principles of patient consent, data confidentiality, and equity in health resources distribution [57]. Decision makers must continue to find the right balance between confidentiality and sharing information among multiple settings to optimize care and outcomes [61].

Participants noted that planning, and developing an individualized services plan, which is an important change of practices, was challenging for them. A systematic review of qualitative literature on barriers to case manager’ roles in various contexts (acute, primary, or long-term care settings and hospital or community-based settings) revealed that it could be challenging for nurse case managers to perform a new unfamiliar role, while continuing to carry out existing duties and responsibilities [18]. Shared care among many primary care providers within a clinic, using an interprofessional approach [62], team composition and interventions matched to patient needs [46], team building activities, and good communication practices among stakeholders [16, 17, 19] could help improve time management and reduce the burden on nurse case managers. Planning time and strategies to develop case management skills, particularly the individualized services plan process, remains crucial [18, 63, 64]. This study found that collaboration between academic experts in case management and clinical nurses, through a community of practice in the first steps of CMI implementation, was effective in preparing nurse case managers for rapidly changing roles [65].

The research team’s role as an external facilitator of the implementation, helping identify and solve problems, had a positive impact on the CMI. The research team helped by providing CMI training, organizing communities of practice for nurse case managers, and by involving patient partners in planning and executing the CMI. This is supported by a pilot study examining the use of external facilitation for implementation of a new intervention targeting a specific population in 20 clinics, where the implementation expert helped identify and solve problems around individual and collective change efforts [66]. Considering local realities and fostering a relationship of trust and reciprocity with health service managers and providers, external facilitation may foster complex practice changes at a modest cost [66], and accelerate the implementation of complex innovations in primary care [67].

### Limitations

A limitation of this study is that patients’ perceptions of barriers to and facilitators of CMI implementation were not included because they were not involved at this early stage. However, patient partner members of the research team were consulted in data analysis meetings and knowledge transfer planning to add their perspectives on the CMI implementation process. Patient partners recommended adopting a culture of patient-centredness, engagement, and collaboration as the foundation for the CMI to be successful. Another limitation was that most participants were women (42/48), which may affect the transferability of these findings to men.

## Conclusions

This study may help decision makers, care providers and researchers who are considering implementation of CMI in primary care by providing better knowledge about first steps of CMI implementation to inform policies and best practices. Future studies to further understanding of the role of facilitation could be helpful to optimally support implementation of such complex innovations in primary care.

## Supplementary Information

**Additional file 1.**

## Data Availability

The datasets generated and/or analysed during the current study are not publicly available because individual privacy could be compromised but are available from the corresponding author on reasonable request.

## Abbreviations

CMI: Case management intervention(s)
ED: Emergency department
EMR: Electronic medical records
