# Supplementary material for: Implementation analysis of a case management intervention for people with complex care needs in primary care: a multiple case study across Canada
Source: BMC Health Serv Res. 2023 Apr 19;23:377. doi: 10.1186/s12913-023-09379-7 (PMC10116737; doi:10.1186/s12913-023-09379-7)
Supplement: Supplementary file 1 — Additional file 1. [file 12913_2023_9379_MOESM1_ESM.docx]

**Additional file 1. Hudon C et al. Interview guide**

Semi-structured interview guide – for case managers, health services managers and focus groups

Version June 2019

**Contextualization**

1. Would you describe the structure of your clinic?

*Probes:*

*How long has it existed?*

*Who are the professionals working there?*

*How many physicians are there? Nurses? Social workers?*

*How would you describe the interprofessional collaboration within your clinic?*

*What is the vision of your clinic, what are the objectives, its values?*

The following questions are specific to **patients with chronic conditions and complex care needs**. Please respond to the questions based on this clientele.

2. Would you describe the services currently offered by your clinic for patients with chronic conditions and complex care needs?

*Probes:*

*What do you know about this clientele and its needs?*

*Do you prioritize the needs of this clientele?*

*What are the barriers and the facilitators to responding to the needs of this clientele?*

*How would you describe the collaboration and partnership between professionals in the follow-up of this clientele?*

*How would you rate the communication within your clinic regarding the follow-up of this clientele?*

*Do any of the professionals at your clinic perform activities related to the case management of patients with chronic conditions and complex care needs? (Explore based on the four components of case management: 1) Evaluation of patient needs and preferences; 2) development and maintenance of an ISP; 3) coordination of services; 4) education and self-management support).*

3. Would you describe the services presently offered by your external partners (hospital, CLSC, community resources, pharmacies, etc.) for patients from your clinic who have chronic conditions and complex care needs?

*Probes:*

*Could you identify your clinic’s external partners involved in the follow-up of this clientele?*

*Could you describe for us the role of these partners in the follow-up of this clientele?*

*Do you think that external partners are responsive to the needs of this clientele and why?*

*What are the barriers and facilitators faced by your external partners in responding to the needs of this clientele?*

*Do any of your external partners perform activities related to the case management of patients with chronic conditions and complex care needs? (for example, at each the four steps of case management: 1) Evaluation of patient needs and preferences; 2) development and maintenance of an ISP; 3) coordination of services; 4) education and self-management support).*

4. Would you describe the relationship of your clinic with external partners (hospital, CLSC, community resources, pharmacies, etc.) in regard to the follow-up of patients from your clinic who have chronic conditions and complex care needs.

*Probes:*

*How would you describe the interorganizational collaboration for the follow-up of this clientele?*

*How would you rate the communication between your clinic and its external partners for the follow-up of this clientele?*

**Ensure that participant discusses the relationship with external partners at the different steps of case management: 1) Evaluation of patient needs and preferences; 2) development and maintenance of an ISP; 3) coordination of services; 4) education and self-management support.*

5. From the point of view of your clinic, what works well in the follow-up of patients with chronic conditions and complex care needs?

*Probes: services integration, external politics, guidelines, communication, collaboration, etc.*

**Ensure that participant discusses the positive points of follow-up for this clientele at the different steps of case management: 1) Evaluation of patient needs and preferences; 2) development and maintenance of an ISP; 3) coordination of services; 4) education and self-management support*

6. From the point of view of your clinic, what does not work as well in the follow-up of patients with chronic conditions and complex care needs?

*Probes: services integration, external politics, guidelines, communication, collaboration, etc.*

**Ensure that participant discusses the negative points of follow-up for this clientele at the different steps of case management: 1) Evaluation of patient needs and preferences; 2) development and maintenance of an ISP; 3) coordination of services; 4) education and self-management support*

The second part of the interview is about your perception of the case management project that will be implemented in your clinic.

7. Based on the information you have so far, what is your understanding of the case management project that will be implemented in your clinic?

*Probes: perceptions, attitudes, importance given to case management by the interviewee.*

**Ensure that participant discusses the different steps of case management: 1) Evaluation of patient needs and preferences; 2) development and maintenance of an ISP; 3) coordination of services; 4) education and self-management support*

8. In your opinion, what changes will case management bring to the services offered by your clinic for patients with chronic conditions and complex care needs?

*Probes: advantages and disadvantages of case management.*

**Ensure that participant discusses anticipated changes based on the different steps of case management: 1) Evaluation of patient needs and preferences; 2) development and maintenance of an ISP; 3) coordination of services; 4) education and self-management support*

9. Which elements of case management do you perceive as:

- Most adaptable to your clinic?

- Most difficult to implement?

**Ensure that participant identifies elements from the different steps of case management: 1) Evaluation of patient needs and preferences; 2) development and maintenance of an ISP; 3) coordination of services; 4) education and self-management support*

10. As a (interviewee’s profession), how would you describe your capacity to participate in the implementation of case management?

*Probes: capacity of individuals to execute individualized services plans (ISPs), knowledge and comfort with case management, etc.*

11. How would you describe the level of preparation of your team members to participate in the implementation of case management?

*Probes: individuals’ stage of change as they progress toward skilled, enthusiastic, and sustained use of the intervention.*

12. What can you tell me about the main activities that went into planning the implementation of case management and engaging key individuals to drive the project? *Probes: implementation management, information systems; planning in advance; quality of approach.*

*Who are the key actors in the project?*

*Why were they chosen?*

*What was done to select the key actors for the project?*

13. Would you tell us about what is planned for the evaluation of:

- The implementation of case management?

- The experience of individuals (patients, professionals and managers)?

*Probes: reflecting & evaluating; quantitative and qualitative feedback about progress and quality of implementation; regular personal and team debriefing.*

18. Are there other topics that were not discussed that you would like to address?

Thank you for your collaboration.
